# Editorial: The developments of metal-based agents against lung cancer

**DOI:** 10.3389/fphar.2022.1101890

**Published:** 2022-12-08

**Authors:** Yi Gou, Li Liu, Hong Liang

**Affiliations:** ^1^ Laboratory of Respiratory Disease, Guilin Medical University, Guilin, China; ^2^ University of Texas Southwestern Medical Center, Dallas, TX, United States; ^3^ Guangxi Normal University, Guilin, China

**Keywords:** lung cancer, anticancer, metal-based agents, design, activity evaluation

Lung cancer is a major public health concern globally, with approximately 1.8 million people diagnosed and 1.6 million people succumbing to this disease annually (Sung et al., 2021). Moreover, the 5-year survival rate is lower than 17 percent (Hirsch et al., 2017). Therefore, the research and development of anti-lung cancer agents is critical. Metal-based agents have several potential advantages over organic-based agents, such as multiple available redox states, several coordination geometries and numbers, as well as the intrinsic properties of the metal ion (van Rijt and Sadler, 2009; Barry and Sadler, 2013). Therefore, metal-based agents provide more opportunities for the development of anti-lung cancer agents compared to organic agents. This topic “*The Developments of Metal-Based Agents Against Lung Cancer*” reports on the recent advances in metal-based agents for treating lung cancer, thereby providing a new perspective on the treatment of lung cancer.

Although platinum- and ruthenium-based agents have been successful in clinical trials or clinical use, serious side effects and drug resistance have hindered their use (Gou et al., 2021). Stimuli-responsive Pt- and Ru-complexes are effective strategies for reducing side effects and improving clinical efficacy. Furthermore, they allow targeted delivery of Pt/Ru-complexes and activation at the target site, which aids in regulating their efficacy and avoiding side effects. Zhang et al. systematically reviewed the representative (exo-stimuli-responsive, endo-stimuli-responsive, and dual-stimuli-responsive) stimulant-responsive platinum- and ruthenium-based agents utilized in lung cancer therapy. They described various representative examples of stimulus-responsive Pt/Ru-complexes in terms of structural design, response mechanisms, and potential medical applications. They also analyzed the challenges these responsive Pt/Ru-complexes face, such as the need for precise control and initiation of the response system, the requirement for exploring new therapeutic targets and their druggability, and systemic toxicity. Nanocarrier-based combination chemotherapy can reduce the side effects of Pt-based agents. Fang et al. summarized recent progress made in the field of nanocarriers (such as carbon nanohorns, liposomes, and mesoporous silica nanoparticles) containing Pt-based agents and other drugs for the combined treatment of cancer, including lung cancer. Moreover, the challenges and future opportunities in this field are also discussed.

Since the discovery of auranofin’s anticancer ability in 1985, gold anticancer agents have been developed and extensively studied. Zhang et al. comprehensively summarized gold(I)-NHC, gold(I)-sulfur, gold (I/III)-phosphine, gold (III)-(C^N), gold (III)-(C^N^C), and gold (III)-(N^N) complexes for lung cancer treatment. They discussed the anticancer activity and the most likely mechanism of action of these Au(I/III) complexes as anti-lung cancer agents. They also briefly analyzed methods to improve the efficacy of gold complexes against lung cancer.

Iridium is stable at various oxidation states, and its +3 complexes are widely used in medical applications. Ir(III)metal ions are kinetically inert under physiological conditions and are ideal for drug design because their complexes can reach the target without modification (Gou et al., 2021). Yang et al. systematically reviewed the recent progress in Ir(III) complexes against lung cancer, and 70 iridium (III)-based complexes demonstrated good anti-lung cancer activity. Apoptosis, autophagic pathway, induction of immunogenic cell death, inhibition of lung cancer cell migration and cycle arrest are also highlighted as crucial anticancer mechanisms.

Thiosemicarbazone, an important Schiff base, can be coordinated with various metals to form complexes with excellent anti-cancer activity. Bai et al. provided an in-depth review of thiosemicarbazone-copper, -ruthenium, -nickel, -platinum, -gold, -silver, -palladium, -bismuth, -chromium, and -cobalt complexes for lung cancer treatment. They summarized the anti-lung cancer activity and most likely anticancer mechanisms of these thiosemicarbazone metal complexes and discussed their potential as anti-lung cancer agents.
